# Insights into the genome architecture and evolution of Shiga toxin encoding bacteriophages of *Escherichia coli*

**DOI:** 10.1186/s12864-021-07685-0

**Published:** 2021-05-19

**Authors:** Graça Pinto, Marta Sampaio, Oscar Dias, Carina Almeida, Joana Azeredo, Hugo Oliveira

**Affiliations:** 1 CEB - Centre of Biological Engineering, University of Minho, 4710-057 Braga, Portugal; 2 INIAV, IP-National Institute for Agrarian and Veterinary Research, Rua dos Lagidos, Lugar da Madalena, Vairão, Vila do Conde Portugal

**Keywords:** STEC, Shiga toxin-encoding bacteriophages, Genomes, Clusters

## Abstract

**Background:**

A total of 179 Shiga toxin-producing *Escherichia coli* (STEC) complete genomes were analyzed in terms of serotypes, prophage coding regions, and *stx* gene variants and their distribution. We further examined the genetic diversity of Stx-converting phage genomes (Stx phages), focusing on the lysis-lysogeny decision and lytic cassettes.

**Results:**

We show that most STEC isolates belong to non-O157 serotypes (73 %), regardless the sources and geographical regions. While the majority of STEC genomes contain a single *stx* gene (61 %), strains containing two (35 %), three (3 %) and four (1 %) *stx* genes were also found, being *stx2* the most prevalent gene variant. Their location is exclusively found in intact prophage regions, indicating that they are phage-borne. We further demonstrate that Stx phages can be grouped into four clusters (A, B, C and D), three subclusters (A1, A2 and A3) and one singleton, based on their shared gene content. This cluster distribution is in good agreement with their predicted virion morphologies. Stx phage genomes are highly diverse with a vast number of 1,838 gene phamilies (phams) of related sequences (of which 677 are orphams i.e. unique genes) and, although having high mosaicism, they are generally organized into three major transcripts. While the mechanisms that guide lysis–lysogeny decision are complex, there is a strong selective pressure to maintain the *stx* genes location close to the lytic cassette composed of predicted SAR-endolysin and pin-holin lytic proteins. The evolution of STEC Stx phages seems to be strongly related to acquiring genetic material, probably from horizontal gene transfer events.

**Conclusions:**

This work provides novel insights on the genetic structure of Stx phages, showing a high genetic diversity throughout the genomes, where the various lysis-lysogeny regulatory systems are in contrast with an uncommon, but conserved, lytic system always adjacent to *stx* genes.

**Supplementary Information:**

The online version contains supplementary material available at 10.1186/s12864-021-07685-0.

## Background

Shiga toxin-producing *Escherichia coli* (STEC) are important foodborne pathogens, responsible for numerous infections worldwide. STEC infections can progress into serious conditions, such as hemorrhagic colitis or hemolytic-uremic syndrome (HUS), which might lead to the patient’s death [1]. In 2018, there were 8,658 confirmed infections in the European Union, and 11 of the 5,254 known outcomes resulted in the patient’s death, which represent a fatality rate of 0.2 % [2].

STEC strains are characterized by their ability to produce Shiga toxins, considered the major virulence factor of this pathotype. There are two known Shiga toxin types, Stx1 and Stx2, being further divided into three (a, c, d) and nine (a to i) subtypes, respectively [3–6]. The disease outcome is dependent on the Shiga toxin subtype carried by STEC strains, being believed that Stx2a is the most associated with severe forms of the disease [7–11]. Several STEC serotypes have been associated with disease; however, not all are linked with severe infections. The most relevant serotypes in health risk are O157, O26, O45, O91, O103, O104, O111, O113, O145, and O121 [12, 13].

*E. coli* acquires Shiga toxin genes through a lambdoid prophage insertion, known as Shiga or Stx phage. Stx phages are temperate, meaning that their genomes are inserted into the bacterial chromosome upon infection [14]. However, their ability to excise and infect other hosts, that could occur at the gastrointestinal tract [15], makes them important drivers of horizontal gene transfer (HGT) of *stx* genes among *E. coli* serotypes and other members of *Enterobacteriaceae* family [16]. This ability to quickly gain, lose or exchange genes through Stx phages has a high impact on the pathogenicity profile and evolution of STEC strains.

From the early moments, Stx phages have been compared to the Lambda phage, the prototype of Lambdoid phages [17]. Stx phages are known to share similar morphologies, e.g. short or long non-contract tails [16, 18]. Their genomes spanning from 30 to 70 kb, share little homology, although having a similar genetic organization [17]. Only a limited number of genomic studies have been performed so far with Stx phages, usually not including a vast number of genomes, nor considering their hosts (STEC serotypes) and environmental sources [1, 19–22]. Moreover, previous studies demonstrated that the analysis of shared gene content provides a more powerful tool to uncover distant relationships between viral sequences [23–25]. However, this has only been attempted for a small number of STEC Stx phages [26]. Currently, with the advance of sequencing platforms and higher availability of complete STEC genomes, genomic studies can evolve to the new level. For a better understanding of the impact of Stx phages in the STEC ecology, we performed an in-depth genomic study of all available Stx phages, to evaluate their genome diversity and organization, gene composition, as well as their association with specific STEC serotypes.

## Results

### Multiple serotypes carry Stx virulence factors

The well-known heterogeneity of the *E. coli* species is seen in the complete genomes retrieved from the database, with a vast diversity of O and H antigens (additional file 1). From the 787 *E. coli* genome sequences, 179 were identified as STEC, containing one or more *stx* genes. Within the STEC group, several strains belong to the O157:H7 serotype (*n* = 48, 27 %). This is not surprising since this serotype has been regarded as the most problematic in the context of STEC-associated foodborne infections, which has probably triggered a special attention on genomic studies for these strains. The high prevalence of this serotype was followed by O26:H11 (*n* = 13, 7.3 %), O111:H8 (*n* = 13, 7.3 %), O104:H4 (*n* = 13, 7.3 %), O145:H28 (*n* = 7, 3.9 %), O121:H19 (*n* = 6, 3.4 %), O113:H21 (*n* = 4, 2.2 %) and O177:H25 (*n* = 4, 2.2 %). The other 50 serotypes are less represented (≤ 2.0 %).

**Fig. 1:**
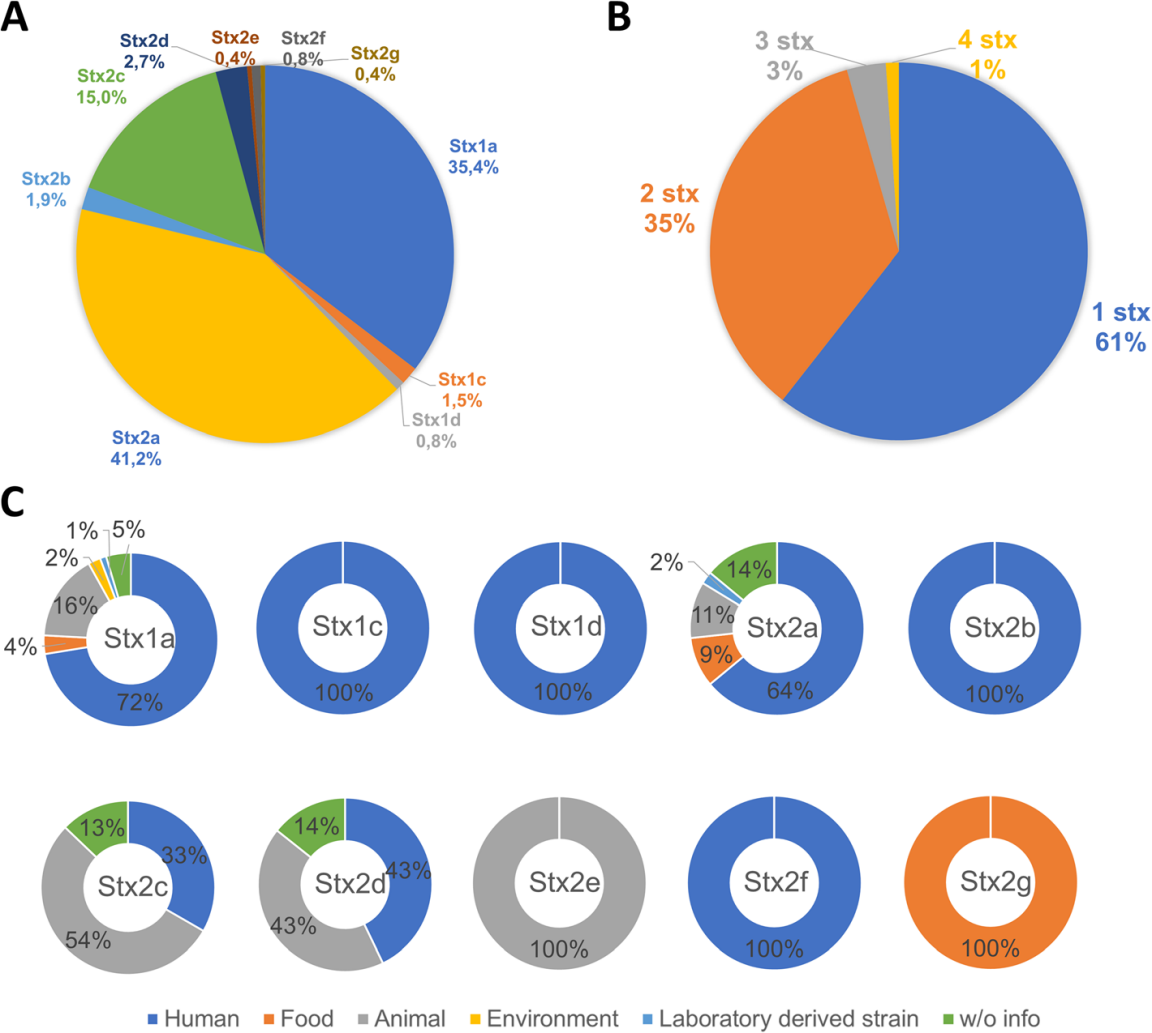
Distribution of *stx* genes. **a** Percentage of each Shiga toxin subtype/variant throughout the *E. coli* dataset. **b** Number of *stx* genes in a *E. coli* strain. **c** The sources were sorted into human, food, animal, environment (creek), mutant, and when no information was available as w/o info. Information collected from the GenBank database for each STEC strain

STEC genomes carrying *stx2* genes are more common (*n* = 161/179, 90 %) than those carrying *stx1* genes (*n* = 100/179, 56 %). Of all *stx* gene variants detected in the *E. coli* dataset, the most common is the *stx2a* (*n* = 106/260, 41 %), followed by *stx1a* (*n* = 93/260, 35 %), and *stx2c* (*n* = 39/260, 15 %), whereas the remaining variants are less prevalent (≤ 2.7 %) (Fig. 1a). Strains carrying only one *stx* gene are the most common (*n* = 109, 61 %), followed by strains carrying two *stx* genes (*n* = 63, 35 %). Moreover, 4 % of the bacterial genomes carry at least three *stx* genes (Fig. 1b). Curiously, one strain carries four *stx2a* genes. The most common combinations of *stx* genes are *stx1a*/*stx2a* (*n* = 35, 20 %), *stx*2a/*stx*2c (*n* = 12, 6.7 %) and *stx*1a/*stx*2c (*n* = 9, 5.0 %). Genes *stx2e*, *stx2f* or *stx2g* were not combined with other *stx* variants. Most STEC strains were detected in humans (*n* = 112, 63 %), followed by animals (*n* = 36, 20 %), particularly in cattle (*n* = 23, 13 %) (additional file 1). Predominantly, *stx1a* and *stx2a* gene variants are usually identified in human isolates (*n* = 68/93 (72 %) and *n* = 71/102 (64 %), respectively). Other frequent gene variant in the database, *stx2c* can be found mostly in animals (*n* = 21, 54 %) followed by humans (n = 13, 33 %) (Fig. 1c). It was confirmed that all STEC genomes had their *stx* genes in intact prophage regions (except for two in questionable prophages), as classified by PHASTER. This program classifies prophage regions as intact (score above 90), questionable (score between 60 and 90) or incomplete (score less than 60). The score calculation is based on the number of genes related to a known phage and the presence of specific genes (coding proteins involved in phage structure, DNA regulation, insertion, and lysis) [27].

### Stx phages can be grouped in four clusters, three subclusters and one singleton

**Fig. 2:**
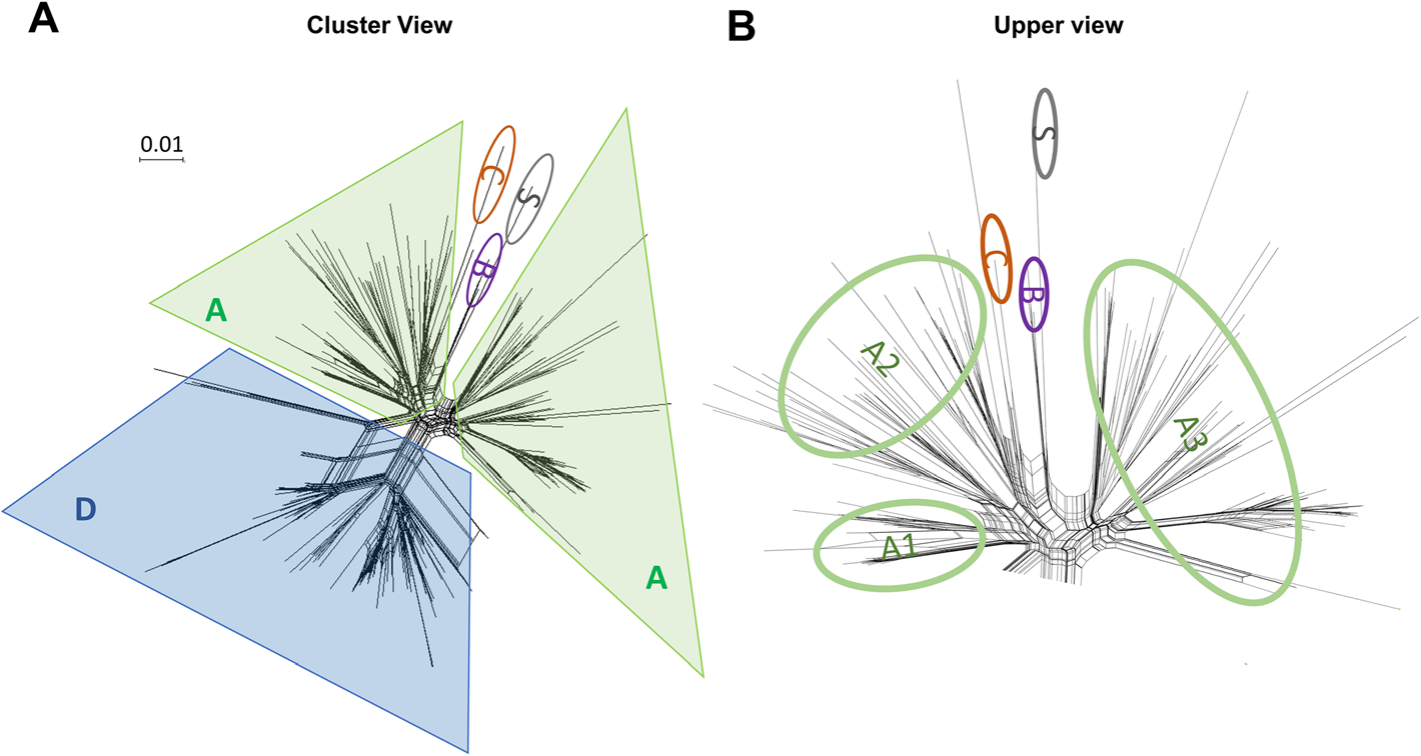
Diversity of STEC Stx phage genomes. The diversity of the 279 STEC stx phages was analyzed converting the shared proteins (phams) into a distance binary matrix using a python script and visualized in Splitstree (3D representation into 2D space). Clusters (**a**) and subclusters (**b**) are divided by colored circles (Green is Cluster A (subcluster A1 to A3), Purple is Cluster B, Orange is Cluster C, Blue is Cluster D, and Grey is Singleton). The scale bar indicates 0.01 substitution

**Fig. 3:**
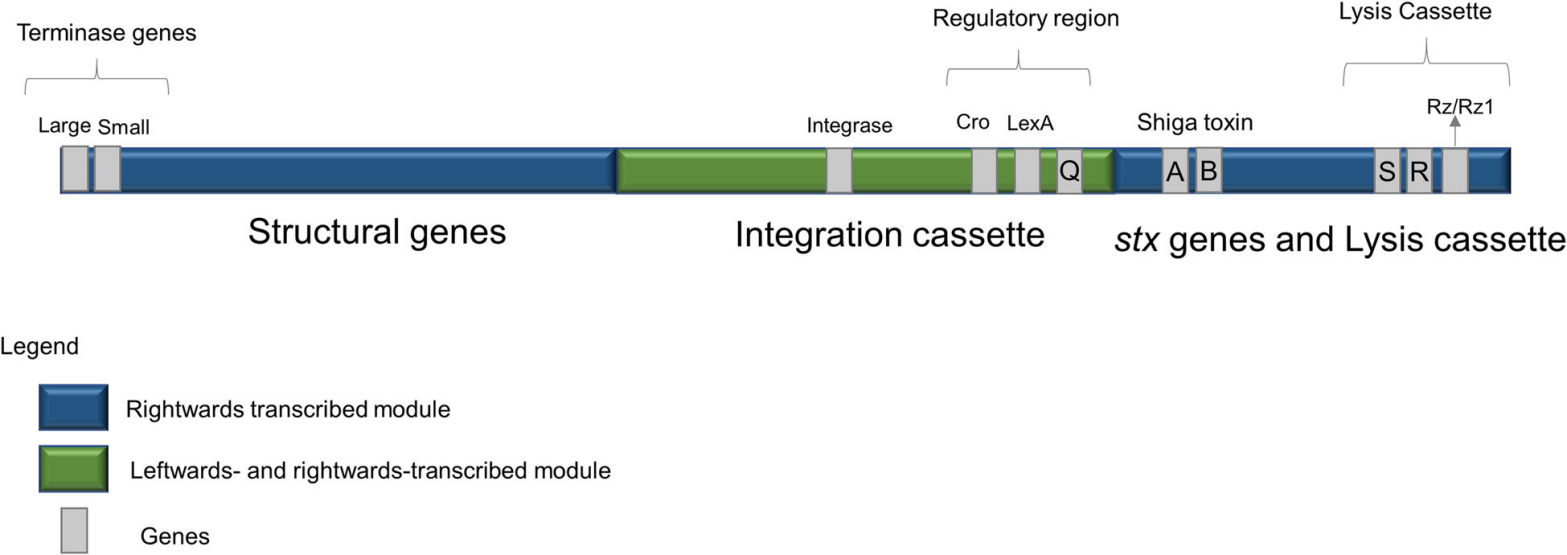
Common genomic structure of Stx phages. To maintain consistency, all genomes were set to start at their terminase genes. Three general modules are identified. The first is related with DNA-packaging and morphogenesis (e.g. capsid, tails, packaging genes); The second is composed by the integration cassette and regulatory genes (e.g. *lexA*, *Cro*, *Q* antiterminator); The third is composed by the virulence (Shiga toxin A and B genes) and the lytic cassette (holin (*S)*, endolysin (*R*) and i-spainin/o-spanin (*Rz/Rz1*) genes)

A dataset of 279 Stx phages were retrieved from the previous group of STEC genomes as of September 2019 (additional file 2) to compare the genomic features of Stx phages. We noted that the genome sizes vary from 31 to 122 kb, containing between 38 and 179 predicted genes. Stx phages were detected in STEC strains with a known *in silico* determined O antigen (46 different O antigens) (additional file 2). Comparative analysis of all Stx phage genomes sorted 24,970 predicted genes into 1,838 phamilies (phams) of related sequences, of which 677 possess only a single sequence (orphams) (additional file 3). As expected, the most conserved phams coded for functions related to the Shiga toxin protein which is composed by a single 30 kDa subunit A and a pentamer of 70 kDa subunits B [28]. Shiga subunit A (pham 538) is present in all Stx phages, and Shiga subunit B (pham 1015) is missing in only one Stx phage (the gene coding for Shiga subunit B has been deleted on the phage KF030445) (additional file 2). Other conserved phams are related to: a Rz i-spanin (pham 1301) present in 277 Stx phages, an SASA family carbohydrate esterase (pham 383) present in 258 Stx phages, and a late gene Q regulator (pham 1422) present in 216 Stx phages. Based on the average shared gene content, Stx phage genomes are grouped into four clusters (A to D), three subclusters (A1 to A3) and one singleton (with no close relatives) (Fig. 2 and additional files 5,6, 7, 8, 9, 10, 11). Generally, all genomes are organized into a rightwards-transcribed left arm containing structural genes, a central leftwards- and rightwards-transcribed integration cassette, and a rightwards-transcribed right arm coding for *stx* genes adjacent to the lysis cassette. The Fig. 3 represents a typical genomic organization of Stx phages. The central block is the most diverse within the clusters.

#### Cluster A – a diverse group of siphovirus-like phages

Cluster A is the largest group (*n* = 159/279, 57 %), subdivided into three subclusters (additional file 4). Members of this cluster vary considerably in genome size (31–119 kb), in predicted number of genes (38–179) and in shared gene content (18–100 %, mean of 34 %). Therefore, cluster A is formed on the basis of Stx phage genomes sharing a meaningful (> 35 %) gene content to at least one member present in cluster A. Most members are predicted siphoviruses, except eight phages for which the virion morphology was impossible to predict (additional file 2). The genomes of cluster A members are organized according to the three major blocks mentioned above. Again, these blocks contain structural genes, integration cassette, and the lysis cassette together with *stx* gene (additional file 5, 6, 7). They were found to contain *stx1* genes (n = 72, 45 %), being all of *stx1a* variants or *stx2* genes (*n* = 87, 55 %), divided into five variants: *stx2a* (*n* = 34), *stx2b* (*n* = 4), *stx2c* (*n* = 40), *stx2d* (*n* = 8) and *stx2g* (*n *= 1) (additional file 2).

Subcluster A1 members (*n* = 22/159, 14 %) (additional file 5) have a wide pairwise shared gene content (42–100 %, mean of 67 %) (additional file 4) and are detected in O111 (*n* = 12), O117 (*n* = 1) and O157 (*n* = 9) strains, isolated from different sources (human, animal and food), in several countries (additional file 2). Two integrase genes were identified, the site-specific integrase (pham 57), and the integrase IntS (pham 1521). In both cases the integrase transcription is leftwards. The position of transposase gene within the genomes is not always the same. All phages carry the same Q regulator (pham 1422) (additional file 3). Subcluster A2 (*n* = 50/159, 31 %) (additional file 6) is the most diverse group, with a wide pairwise shared gene content (15–90 %, mean of 39 %) (additional 4). Host strains from this group present a great diversity of O-antigens (*n* = 24), and are mostly isolated from human sources (*n* = 47/50, 94 %) (additional file 2). Integrases were either of tyrosine-type integrase (phams 1704, 1590 or 797) or site-specific integrase (phams 57, 23, 1536, 536 or 535) transcribed in both directions. No transposase genes were identified. In this subcluster several genes coding for Q regulator were found (pham 1422, 528, 984, 1414). Subcluster A3 (*n* = 87, 55 %) is the largest group of phages within cluster A (additional file 7), with shared genes ranging between 23 and 100 % (mean of 50 %). The strains O-antigens were also quite diverse (n = 20), with two strains with unidentified O-type (additional data 2). Integrase genes are diverse (site-specific integrase versus tyrosine-type recombinase/integrase) that can either be transcribed leftwards or rightwards. There are phage genomes that carry more than one integrase gene. In this subcluster several genes coding for Q regulator were found (pham 1422, 978, 984 or 528).

#### Cluster B – a distinct group of siphovirus-like phages

Cluster B (n = 3/279, 1 %) is a small group composed of three predicted siphoviruses (additional file 2) with high shared gene content (72–86 %, mean of 85 %) (additional file 4). Their genomes are relatively small (39–51 kb), with 61 to 80 predicted genes (additional file 8). All three Stx phages were detected in strains with different O-antigen (O26, O63 and O145) from different sources (pigeons and human). Curiously, cluster B members are the only ones containing the *stx2f* gene variant (additional file 2). While the beginning and end of the three genomes are similar, the middle module is the most diverse, especially for phage LN997803, as seen by the purple lines (no shading reflets the lack of DNA similarity below the cut-off of 10^− 4^ of BlastN [29], additional file 8). Interestingly, although they contain several integrases, pham 536 is present in all three phages. As seen in other clusters, there is more than one transposase within each genome.

#### Cluster C – myovirus-like representatives

Cluster C (*n* = 2/279, 0.72 %) is the smallest group (additional file 9) with a predicted myovirus and an unclassified phage, with relatively low shared gene content (35 %). The genomes vary in size (43–78 kb), and therefore vary in the number of predicted genes (66 vs. 114) (additional file 2). Stx phages carry the *stx2e* gene variant, the only ones found in the dataset. These phages were detected in strains of different O-antigen strains (non-identified and O116), and the source and country of isolation are also different, from a human in Germany and from a pig in China, respectively. The most conserved phams between these phages are the ones responsible for the lysis-lysogeny decision (CI and LexA proteins), Shiga toxin and the lysis cassette. The Q regulators are different (pham 1422 vs. pham 1424).

#### Cluster D – a big group of close podovirus-like phages

Cluster D (*n* = 114/279, 41 %) contains a vast number of genomes of predicted podoviruses, with a wide shared gene content (25–100 %, mean of 55 %) (additional file 4). Some phages have an even lower shared gene content with some members, due to the lack of the first module (phams related with structural proteins) (additional file 10). The genome size ranges from 37 to 122 kb, however, most fall between 60 and 81 kb (*n* = 103). The number of predicted genes is between 64 and 161. Phages were detected in several strains of different O-antigens (*n* = 25), and from two strains of *Shigella* spp. (*S. flexneri* 2a and *S. sonnei* 75/02 were included as representatives of Stx phages detected in other close related species [30]), being isolated from a wide range of sources and countries. Their genomes have either *stx1* genes (*n* = 24, 21 %), divided into *stx1a* (*n* = 22), *stx1c* (*n* = 1) and *stx1d* (*n* = 1); or *stx2* (*n* = 88, 77 %), divided into *stx2a* (*n* = 62), *stx2c* (*n* = 2), *stx2i* (*n* = 1). The differentiation of the remaining *stx2* genes was not possible (additional file 2). Phages have either the site-specific integrase transcribed leftwards (phams 57 (the most common), 1536 or 535) or a combination of integrase IntS transcribed leftwards (pham 1521) with site-specific integrases transcribed rightwards (pham 1536). The number of transposases varies considerably, with some genomes having one or more copies transcribed in opposite directions (e.g. phams 238, 154, which seems to appear always in combination). Other phage genomes have only one transposase transcribed leftwards (pham 238) after the *stx* gene. The Q antiterminator is the most common regulator identified in our dataset (pham 1422). For most members (*n* = 108), the first module is similar (as depicted by the purple lines in map of additional file 10). However, some phages have differences in the tail genes. We noted that within this cluster, there are two podoviruses and one unclassified phage (STX2A_CP027445.1_2, STX2A_CP027459.1_10, STX2A_CP013029.1_11) with considerably lower shared gene content (28 %) (additional file 4), that have two blocks with predicted genes for the lytic cassette (additional file 10). This phenomenon is also observed for phages in other clusters.

#### Singleton – a very singular phage

The singleton identified (STX1D_CP027447.1_5) (additional file 2) shares fewer than 28 % genes to any of the Stx phage genomes in the dataset. Its genome has a size of 79 kb with 119 predicted genes, one of them being the *stx1d*. About 48 predicted genes are orphams, being the remaining phams present in cluster A genomes. The genome structure is similar to the other phages (as represented in Fig. 3), having several integrases annotated, but no transposase was identified (additional file 11).

### The regulatory region structures for lysis-lysogeny decision are diverse

Stx phages are known to be lambdoid phages for which several mechanisms for prophage induction have been identified [31]. In our study it was possible to identify a vast number of phams related to the lysis-lysogeny regulation, as Q antiterminator protein [21], LexA regulator [32], antirepressor Ant [33] or Cro/CI repressor proteins [34]. The phams that have a conserved domain are shown in Table 1. As observed in other studies [35], the regulatory region is located upstream of the *stx* genes and the lysis cassette (additional files 5, 6, 7, 8, 9, 10, 11). Overall, it seems that phages within the same cluster (or subcluster) share the same organization.

The Q regulator is an important member of the regulatory region of Stx phage genomes [21]. Indeed, several phams were annotated with this function, being the most conserved: pham 1422 (present in 216 phages of multiple clusters), pham 978 (present in 29 siphoviruses), and the pham 528 (present in 18 siphoviruses and unclassified phages). Another known gene of this region is the repressor *lexA*, for which there are six phams identified, all with the same domain (COG1974). The most common is pham 257 (present in 116 phages of all clusters), followed by pham 237 (present in 15 siphovirus and unclassified phages of cluster A). Unexpectedly, for some phages, this regulator was not detected. LexA is normally transcribed leftwards and present once in the genome (additional file 3). Nevertheless, few phages have two different phams related to LexA functions (phams 1059 and 237). Repressors Cro and CI are important members that regulate the lytic excision of the prophage [36] by self-regulating its promoter, inhibiting the expression of the all others genes of the prophage [33]. Several phams (n = 18) with these regulatory properties were identified (Table 1), usually transcribed rightwards as the Q antiterminator phams (Table 1, additional file 3). The antirepressor Ant is an important gene of the superinfection ability of P22 phage [33], being present in 126 Stx phage genomes (phams 649 and 629 in 89 and 35 members, respectively) (Table 1).

### The conserved lysis cassette is located upstream the ***stx*** genes

The prototype phage Lambda genome is known to have a lysis cassette composed of the *S* (holin), *R* (canonical endolysin) and *Rz/Rz1* (i-spanin/o-spanin, being the latter embedded in the + 1 reading frame of *Rz*) genes [37]. In our dataset, the endolysin gene is conserved (phams 858, 838 and 762, shared by 225, 44 and two phages, respectively). Both phams 858 and 838 are predicted SAR (signal-arrest-release) endolysins having an N-terminal R21-like domain and present in all clusters (except cluster C). On the other hand, pham 762 is predicted to be a canonical endolysin, having a metallopeptidase domain without a signal peptide, and exclusively present in cluster C members. The holin function is split in more genes (phams 52, 108, 135, 374, 891 and 1132, in which the latter two are the most conserved, present in 138 and 72 phages, respectively). Conceivably, this protein functions as a pin-holin (in phams 108, 135, 374, 891 and 1132) and is associated with a predicted SAR endolysin or acts as a classical holin (in pham 52) when associated with canonical endolysins (exclusively found in cluster C). The *Rz/Rz1* (pham 1301) is one of the most conserved genes among the genomes of the prophages analyzed (present in 277 phages), only surpassed by the genes coding for Shiga toxin subunits A and B (pham 538 and pham 1015, respectively), present in all phages. The embedded *Rz1* o-spanin is not represented in the genomics maps due to the automatic annotation of the phage genomes. The lysis cassette map of some phages is represented in additional file 12. The holin was not found in 11 Stx phages, of which 10 phages (from subcluster A1) only have the i-spanin (pham 1301) identified (with no endolysin or holin detected) (Additional file 5)

**Table 1:** Lysis-lysogeny related phams identified in the Stx phage genomes

Pham	# Phage	Function	Domains
78	7	Helix-turn-helix transcriptional regulator (Cro/C1-type HTH and peptidase s24 domains)	COG2932
87	4	Helix-turn-helix transcriptional regulator Cro and CI	pfam01381
115	3	Helix-turn-helix transcriptional regulator Cro and CI	pfam01381
124	2	Repressor LexA	COG1974
237	15	LexA family transcriptional regulator	pfam01726
257	116	LexA family transcriptional regulator	COG1974
393	15	Helix-turn-helix transcriptional regulator (Cro/C1-type HTH and peptidase s24 domains)	COG2932
507	22	Transcriptional regulator Cro and CI	pfam01381
528	18	Antitermination protein	pfam03589
603	3	Helix-turn-helix transcriptional regulator Cro and CI	pfam01381
629	35	Phage antirepressor Ant	COG3561
641	13	Regulatory protein CII	pfam05269
649	89	Phage antirepressor Ant	COG3561
837	3	Helix-turn-helix transcriptional regulator Cro and CI	pfam01381
874	1	Helix-turn-helix transcriptional regulator Cro and CI	pfam01381
901	138	Hypothetical protein (CII regulatory)	pfam05269
907	5	Transcriptional regulator Cro superfamily	COG4197
921	22	Helix-turn-helix transcriptional regulator (Cro/C1-type HTH and peptidase s24 domains)	COG2932
922	9	HTH-type transcriptional regulator RdgA (Cro/C1-type HTH and peptidase s24 domains)	COG2932
968	1	Antiterminator Q	pfam06530
978	29	Antiterminator Q protein	pfam06530
984	13	DUF1133 family protein (Q antiterminator HHPred)	cl29970
1009	8	Cro/Cl family transcriptional regulator	COG4197
1010	1	Rha family transcriptional regulator Cro superfamily	COG4197
1059	7	LexA family transcriptional repressor	COG1974
1199	1	Transcriptional regulator lambda repressor-like DNA-binding domain	COG3423
1204	2	LexA family transcriptional regulator	COG1974
1204	2	LexA family transcriptional regulator	COG1974
1318	11	Helix-turn-helix transcriptional regulator Cro and CI	pfam01381
1392	1	Helix-turn-helix transcriptional regulator Cro and CI	pfam01381
1422	216	Late gene regulator Q	pfam06530
1424	7	Antitermination protein	pfam06530
1457	2	LexA family transcriptional regulator	COG1974
1550	1	Antiterminator Q	pfam06323
1642	2	Transcriptional regulator Cro protein	pfam14549
1673	3	Helix-turn-helix transcriptional regulator Cro and CI	pfam01381
1763	64	Helix-turn-helix transcriptional regulator (Cro/C1-type HTH and peptidase s24 domains)	COG2932
1266	1	Phage antirepressor Ant	pfam03374
1639	16	Putative antirepressor	pfam03374
1668	1	Phage antirepressor Ant	COG3561

## Discussion

This study compared 787 complete *E. coli* genomes available at GenBank database as of September 2019. Their genomes were typed for their antigens (O and H-antigens), using the Web tool SerotypeFinder [38], and *stx* genes, using the Web tool VirulenceFinder [39]. Additionally, a dataset of 279 Stx phages was curated and their genomes grouped into clusters based on gene content similarity. The dataset was constructed through PHASTER, an online tool able to predict prophage regions within a bacterial genome [40]. Additionally, 55 Stx phage genomes (including two of *Shigella* spp., reported to be similar do STEC Stx phages [41, 42]) were directly retrieved from the GenBank and added to our dataset.

For several years, STEC strains were classified using a simple scheme of O157 and non-O157 [43], as this STEC serotype has been considered the most pathogenic, making it the most extensively studied in reference laboratories worldwide [44]. Recently, other serotypes have also been recognized as important pathogens, as reflected in STEC detection standards used nowadays (the technical specification of the International Organization for Standardization ISO/TS 13,136:2012 and Microbiology Laboratory Guidebook 5 C.00 from United States Department of Agriculture). Indeed, we found the O157:H7 to be the most represented serotype (26.8 %); but others, such as O104:H4 and O26:H11, were also highly represented in our dataset (both around 7 %), which can be explained by recent outbreaks where new STEC serotypes have been continually emerging [14, 45]. Regarding the Shiga toxins, three subtypes for Stx1 (a, c, and d) and nine for Stx2 (a to i) are currently known [3–6]. Different studies have shown that Stx2a is the most virulent, being often associated with HUS. However, Stx1a, Stx2c or Stx2d toxins are also associated with the development of HUS [46]. As expected, the subtype more detected in humans was the Stx2a, either alone or associated with Stx1a or Stx2c (additional file 1). Several subtypes, namely Stx2e-g, were less represented (Fig. 1a), which can be explained by their inherent low virulence, and therefore rarely detected. In fact, most strains (63 %) included in our dataset were isolated from humans, of which 21 % were from confirmed illness cases (additional file 1).

We found all *stx* genes within intact Stx phages (except for two questionable prophages), rendering them the ability to potentially excise and infect new hosts, as observed in *Shigella* and *Aeromonas* strains, which carry Stx phages homologous to the ones detected in STEC [41, 47]. We also found a significant portion (39 %) of STEC strains with more than one Stx phage (Fig. 1b). Fogg et al. (2011), demonstrated that phage Φ24_B_ could insert itself multiple times on the same host [33]. The integrase, outside of the regulatory control of phage repression region, could be the responsible for this behavior. As the integrase is constantly expressed, this would allow the prophages to be established. Similarly, in our study, multiple integrases transcribed outside the regulatory region were also observed, e.g. Subcluster A1 (additional file 5), which could also explain multiple infection events.

These Stx phages’ ability to infect distinct serotypes, even from different pathotypes, can lead to serious global health outcomes. A good example are the Stx2a-converting phages infecting *E. coli* O104:H4, a known Enteroaggregative *E. coli* (EAEC) responsible for the outbreak of 2011 in Germany and other European countries [45, 48], which became known as a novel hybrid pathotype (EAEC/STEC) [48]. In our dataset, 14 predicted podoviruses were similar, sharing 88 % of its genes to the Stx2a-converting phage isolated during the 2011 outbreak (additional files 2, 4 and 10). Moreover, a similar high shared gene content between 81 and 100 % is observed to others prophages detected in different serotypes, as O2:H27 isolated from cattle [49], O111:H8 isolated from human [50] and O168:H8, isolated from food [51]. This suggests the ability of some prophages to exchange hosts with different serotypes. Beutin et al. hypothesis that STEC strains not commonly associated with human disease can work as the source of Stx2a-converting phages, able to convert EAEC into a high virulent strains [49], is in agreement with our analysis that identified 13 EAEC/STEC strains.

Through multiple lines of evidence, the STEC Stx phages were shown to be extremely diverse. First, their genome sizes ranged between 30 and 122 kb, broader than previously anticipated in 2015 (30–70 kb) [17]. Of note, some STEC genomes appeared to have prophages inserted in series, which can be an artefact from PHASTER that failed at recognizing the genomes ends of prophages in close proximity. The apparent increased size, comparatively to the archetype phage lambda (45.5 kb), suggests that the additional genes acquired serves to modulate their lysogens rather than being involved in the phage core functions *per se*. Some phams identified in these longer genomes, are related to metabolism, such as D-serine ammonia-lyase (pham 184), Exodeoxyribonuclease VIII (pham 1056) and Multidrug efflux MFS transporter permease subunit EmrY (pham 1610), being inserted in the middle module. Second, the genomes could be grouped into four clusters and one singleton based on shared gene content similarity, although even within the clusters, a vast range of shared gene values was observed.

Interestingly, cluster assignment is in agreement with their predicted virion morphologies, i.e. *Siphoviridae* (clusters A-B), *Myoviridae* (cluster C) and *Podoviridae* (cluster D). The fact that phage genomes from clusters B and C exclusively code rare (or underestimated) toxins (*stx2f* and *stx2e*, respectively) from a pool of 12 detected variants, is a significant trend that warrant further investigation. Third, besides generally being organized into three major modules coding for morphogenetic (rightwards-transcribed); integration cassette (transcribed both ways) and lysis cassette functions (rightwards-transcribed), the genomes have frequent small non-homologous modules and synteny breaks between genomes, similar to what was observed in phage genomes infecting other hosts (e.g. other *Enterobacteriaceae, Staphylococcus*, *Pseudomonas, Mycobacterium*) [23, 25, 26, 52]. This high mosaicism pattern can be interpreted as a continuous adaptation of the phage fitness to infect other hosts and/or to survive in several environments likely driven by HGT. Finally, the vast diversity of Stx phages is also seen at the gene level. From a total of 20,382 predicted genes, 1,838 families could be sorted using Phamerator. Most (78 %) were shared by ten or fewer Stx phages, with a significant percentage (37 %) being unique genes (displayed in genome maps as white boxes). The fact that this percentage of single gene lies in close proximity of other phage populations infecting hosts with higher taxonomic levels, yet similar population sizes (e.g. *Staphylococcus* phages have 35 % single gene), is very meaningful. This vast gene diversity is likely driven by continuous gene influx from novel bacterial hosts and/or other phages by HGT and demonstrates an untapped reservoir of genes with potential ecological (e.g. repressor, toxins) and biotechnological (e.g. lytic proteins, recombinases) roles.

Several mechanisms can be responsible for the incorporating genes by prophages, including transposases [52], which are the most abundant genes in nature [53]. The transposase and insertion sequences (IS) are commonly found in bacterial genomes, being responsible for several gene arrangements [54], which modulate the genome evolution [55]. Several transposases and IS elements were identified in our study, including within the same Stx phage genome. Some were found near *stx* genes, either up or downstream. Nevertheless, most transposases were found on the morphogenetic and integration modules (additional files 5, 6, 7, 8, 9, 10, 11). Such mobile elements’ insertion is an important evolutionary element and diversification of the lysogens, since it can introduce new genes into the genomes [56]. It can also result in the regulation of the adjacent genes. Kusumoto et al., in 2000, associated the regulation of Shiga toxin productivity with the integration of an IS1203v element (classified into the IS3 family) adjacent to the *stx2* genes. It was demonstrated that with the IS element’s excision, the ability to express Shiga toxin was regained [54]. As seen in our pham dataset, the IS3 family is the most distributed in STEC Stx phages, present in 109 genomes (additional file 3). Further investigation is needed to fully understand the impact of the insertion and excision of these elements on the evolution, regulation, and perhaps (in)activation of Stx phages.

It is generally accepted that the Shiga toxin production and the transference of *stx* genes are related to the induction and subsequent excision of Stx phages [57]. The phams related to the lysis-lysogeny decision were common for all Stx phages of the dataset (additional file 3 and Table 1), as reported in other Stx phage populations [58–60]. However, several organizations were detected, with different additional genes incorporated adjacent to Cro, cI and Q regulators (additional file 5, 6, 7, 8, 9, 10, 11). The diversity in the regulatory genes’ architecture can explain distinct Shiga toxin expression levels, as well as the strains’ potential to produce phage particles without inducer agents [61]. In fact, spontaneous production of Shiga toxin was previously observed for phage 933W [35]. In opposite to phage Lambda, phage 933W does not form a DNA loop, responsible for connecting adjacent operators, repressing promotors related to the lytic growth. In Bullwinkle et al., the authors described that the concentration of repressor needed to activate or repress the lytic functions is low, explaining in part the spontaneous phage induction. Therefore, phage 933W has evolved to regulate its lysogen through different strategies [34]. To better understand the extent of the different regulatory mechanisms in Stx phages, more in-depth studies are needed. The location of virulence genes downstream of lysis module is common, associating the virulence feature’s expression to phage induction [25]. An important aspect of lambdoid phages is that the integrase genes are regulated by the lysis-lysogeny regulatory region [62]. However, it was reported that, for some Stx phages, these genes are transcribed in opposite directions. This leads to a constant expression of integrases allowing multiples prophages in the same lysogen [33]. In fact, several integrase phams were identified in our database, transcribed in both directions, usually outside the lysis-lysogeny regulatory region.

Regarding with the lytic cassette, that of phage lambda contains a canonical holin that forms large pores exposing the peptidoglycan locally to endolysins in a genetically determined time [63]. However, almost all Stx phages herein analyzed (99 %) have a predicted SAR endolysin and pin-holin. The SAR endolysin N-terminal signal is known to mediate export through the host secretory system, being activated by membrane depolarization performed with the pin-holins, which is believed to make small holes in the host cell membrane [63]. We found the lysis genes to be in close proximity with each other and located downstream the integration cassette and adjacent to the *stx* genes. In-between the *stx* and the lysis genes, there is a region, typically around 3 to 3.5-kb, where several genes are found, which could also have impact their lysogens, therefore warranting further investigation. This hypothesis of having additional virulence genes located downstream the integration cassette would mirror the generic organization of other prophages that infect different hosts (e.g. *Staphylococcus*) [25]. All STEC Stx phage late genes are located downstream and in the same transcriptional orientation of the anti-terminator gene Q (with fewer phams being transcribed in the opposite direction), controlling their expression [64, 65]. We were also able to demonstrate that phage genomes carrying different *stx* alleles share similar Q genes. However, in some cases, the Q gene was incomplete or missing, presumably due to recombination events [66]. The synthesis of *stx* genes is then unequivocally linked to the cell lysis once the STEC Stx phages enter the lytic cycle, as experimentally validated in several studies [67–69]. Nevertheless, there is still a good evolutionary theory to be disclosed to support the discrete location of the virulence factors in Stx phage genomes, as other locations within the late region would serve the same goal.

## Conclusions

There is a considerable diversity of STEC serotypes that do not fall into the well-known O157:H7 and the so-called top six non-O157 serotypes (O26, O45, O103, O111, O121, O145), which carry complete Stx phages with *stx* genes variants. All *stx* genes in STEC strain genomes seem to be phage-borne located in intact prophages, regardless of the phage type, STEC serotype, geographical region, and sample origin. Stx phages were divided into four clusters and one singleton, based on their gene shared content, which is in agreement with their predicted morphologies. Despite the vast regulatory region structures for lysis-lysogeny, the conserved *stx* location in the lytic cassette, strongly suggests a role of *stx* expression during prophage excision, but other alterative mechanisms cannot be discarded without further investigation.

## Methods

The *E. coli* prophage dataset was constructed using complete *E. coli* sequenced genomes (*n* = 787) deposited at GenBank in September 2019. *In silico* typing (O and H antigens) was performed using SerotypeFinder 2.0 (additional file 1) [38], using 85 % as threshold for identity and a minimum length (number of nucleotides a sequence will overlap) of 60 %.

Biopython 32 package was used within the conda environment, and python scripts were used to automatically scan each *E. coli* genomes for prophages through PHASTER API [40]. Prophages were retrieved and identified as incomplete (*n* = 1757), questionable (*n* = 763) or intact (*n* = 4392), depending on the completeness or their potential viability. Each prophage was screened for *stx* genes (*stx1a*, *stx1c-d*, *stx2a-g*, for both subunits A and B) using VirulenceFinder, with standard parameters [70], resulting in 259 intact Stx phages (for incomplete prophages no *stx* genes were found; moreover, only two questionable phages were detected that were not included on the final dataset). The final dataset of 279 Stx phages was constructed using 224 randomly selected intact Stx phages from the previous analysis with 55 Stx phages directly retrieved from GenBank (including two phages from *Shigella* spp. since these Stx phages are also detected in foodborne pathogens [71]) (additional file 2).

For consistency, all Stx phage genomes (from PHASTER or GenBank) were set to start at the terminase genes, and re-annotated using Geneious Prime [72]. Protein functions were manually inspected using BLASTP (blast.ncbi.nlm.nih.gov) against the NCBI nonredundant protein database and the NCBI Conserved Domain Database (CDD) with CD-Search (ncbi.nlm.nih.gov/Structure/cdd). In some cases, they were inferred based on structural similarity using HHpred server with Protein Data Bank database (toolkit.tuebingen.mpg.de/#/tools/hhpred). An E-value cutoff of 1 × 10^− 5^ were used for all searches.

Whole-genome comparisons of Stx phages were made using Phamerator [73], which allowed the analysis of the shared gene content by grouping genes into phams with Kclust algorithm (additional files 3–4) and to generate comparative genome maps using the “Align Two Sequences” algorithm of BLASTN (additional files 5, 6, 7, 8, 9, 10, 11). The shared gene content was visualized in SplitsTree [74]. Phage genomes were assigned into only one cluster when sharing 35 % of shared genes (phams) or as singletons if sharing fewer genes to all members, a metric previously used to assign phage membership [25]. Stx phage morphology was predicted based on the most homolog phage found using BLASTN (blast.ncbi.nlm.nih.gov) [75] using “Tailed phages” (taxid:28,883) database.

## Supplementary Information

**Additional file 1. **E. coli strains information. Serotype and Virulence tab contains the information of all 787 strains, while the Stx carriers tab has only the strains where at least one stx gene is found. For all strains, the O and H antigens, stx genes, origin (source of isolation) and country of isolation is provided, using SerotypeFinder, information retrieved from GenBank or from a reference article (when available). w/o, information not available.

**Additional file 2. **STEC Stx phages information. The information for all 279 Stx phages used in the final dataset is provided. Phages were characterized for: sequence length, number of proteins, cluster, predicted family, similar phage (hit of BlastN, query cover, E-value, percentage identity), Stx subtype/variant, the lysogen O-type, as well as origin (source of isolation) and country of isolation. For those retrieved directly from GenBank database, the reference article is provided. w/o, information not available.

**Additional file 3. **Phams identified in the Stx phages dataset. The dataset includes 279 Stx phages, encoding a total of 24,970 predicted proteins sorted into 1,838 phamilies (phams) of related proteins, 677 of which were identified as orphans (genes without related sequences) using Phamerator.

**Additional file 4. **Shared gene content matrix. Phamerator output (1,838 phams) was converted to a matrix of shared gene content. Heat map was created in excel.

**Additional file 5. **Whole-genome map of subcluster A1 phages. Maps were generated with Phamerator, where pairwise sequence similarity (minimal BLASTN cut-off E value is 10− 4) is given according to color spectrum (purple lines for highest and red lines for the lowest nucleotide similarity, no shading shows no similarity with a BLASTN score of 10-4 or better). Ruler corresponds to genome base pairs. Labelled ORFs with predicted function are shown as colored boxes (white boxes represent orphans, singe genes) position above (rightwards transcribed) or below (leftwards transcribed) the bar. Gene numbering reflects the re-organization of genomes. All genomes were set to start at the terminase genes.

**Additional file 6. **Whole-genome map of subcluster A2 phages. Represented as in Additional file 5.

**Additional file 7. **Whole-genome map of subcluster A3 phages. Represented as in Additional file 5.

**Additional file 8. **Whole-genome map of cluster B phages. Represented as in Additional file 5.

**Additional file 9. **Whole-genome map of cluster C phages. Represented as in Additional file 5.

**Additional file 10. **Whole-genome map of cluster D phages. Represented as in Additional file 5.

**Additional file 11. **Whole-genome map of singleton. Represented as in Additional file 5.

**Additional file 12. **Lysis cassette map. The map was constructed using Phamerator using six randomly Stx phages as an example. Genes are labelled with their putative function, and phams with same predicted functional are represented by same color. Similarity is given by purple lines (minimal BLASTN cut-off E value is 10− 4).

## Data Availability

Data generated and analyzed throughout this study are included in this published article and in the additional information files. *Escherichia coli* complete genomes were retrieved from GenBank database and each accession number can be found in Additional file 1. Prophage’ genomes were retrieved using PHASTER web tool using the *E. coli* genomes’ accession numbers and can be provided upon request.

## Abbreviations

STEC: Shiga toxin-producing Escherichia coli
*st**x*: Shiga toxin
phams: Phamilies
HUS: Hemolytic-uremic syndrome
Stx1: Shiga toxin subtype 1
Stx2: Shiga toxin subtype 2
HGT: Horizontal gene transfer
S: Holin
R: Canonical endolysin
Rz/Rz1: i-spanin/o-spanin, being the latter embedded in the + 1 reading frame of Rz
SAR: Signal-arrest-release
EAEC: Enteroaggregative E. coli
IS: Insertion sequence
