# Supplementary figures and images for: Insights into the genome architecture and evolution of Shiga toxin encoding bacteriophages of Escherichia coli
Source: BMC Genomics. 2021 May 19;22:366. doi: 10.1186/s12864-021-07685-0 (PMC8136144; doi:10.1186/s12864-021-07685-0)

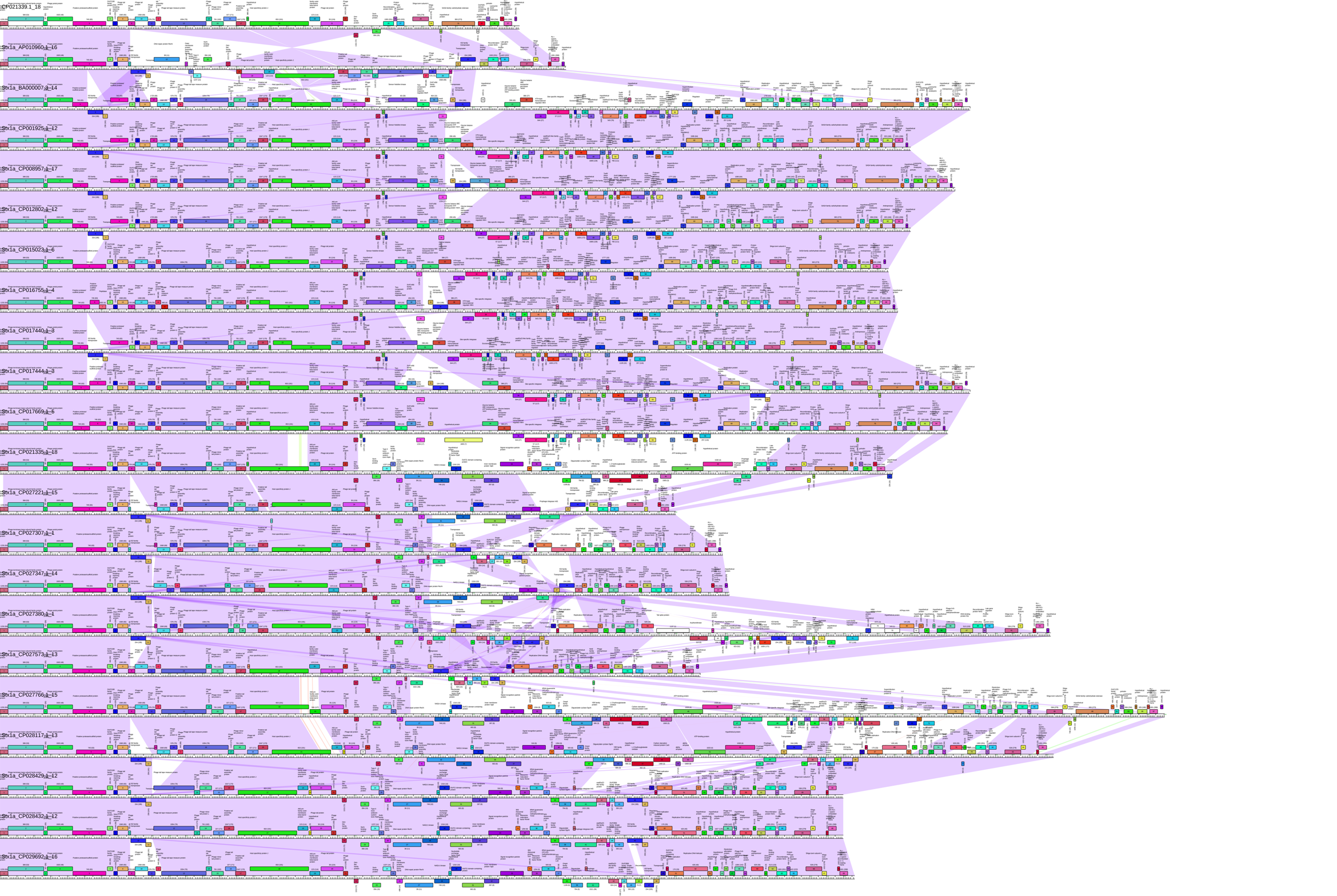

Supplement: Supplementary file 5 — Additional file 5. Whole-genome map of subcluster A1 phages. Maps were generated with Phamerator, where pairwise sequence similarity (minimal BLASTN cut-off E value is 10− 4) is given according to color spectrum (purple lines for highest and red lines for the lowest nucleotide similarity, no shading shows no similarity with a BLASTN score of 10-4 or better). Ruler corresponds to genome base pairs. Labelled ORFs with predicted function are shown as colored boxes (white boxes represent orphans, singe genes) position above (rightwards transcribed) or below (leftwards transcribed) the bar. Gene numbering reflects the re-organization of genomes. All genomes were set to start at the terminase genes. [file 12864_2021_7685_MOESM5_ESM.pdf]

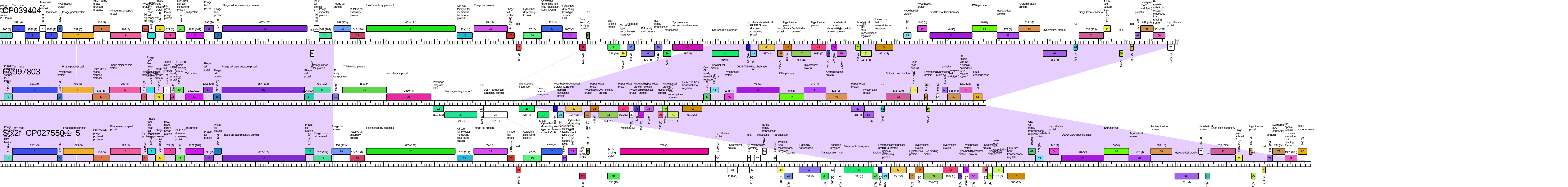

Supplement: Supplementary file 8 — Additional file 8. Whole-genome map of cluster B phages. Represented as in Additional file 5. [file 12864_2021_7685_MOESM8_ESM.pdf]

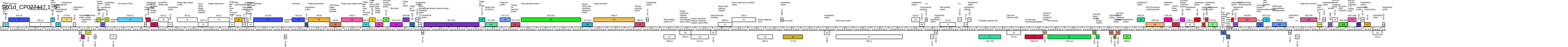

Supplement: Supplementary file 11 — Additional file 11. Whole-genome map of singleton. Represented as in Additional file 5. [file 12864_2021_7685_MOESM11_ESM.pdf]
