# Supplementary material for: Insights into the genome architecture and evolution of Shiga toxin encoding bacteriophages of Escherichia coli
Source: BMC Genomics. 2021 May 19;22:366. doi: 10.1186/s12864-021-07685-0 (PMC8136144; doi:10.1186/s12864-021-07685-0)

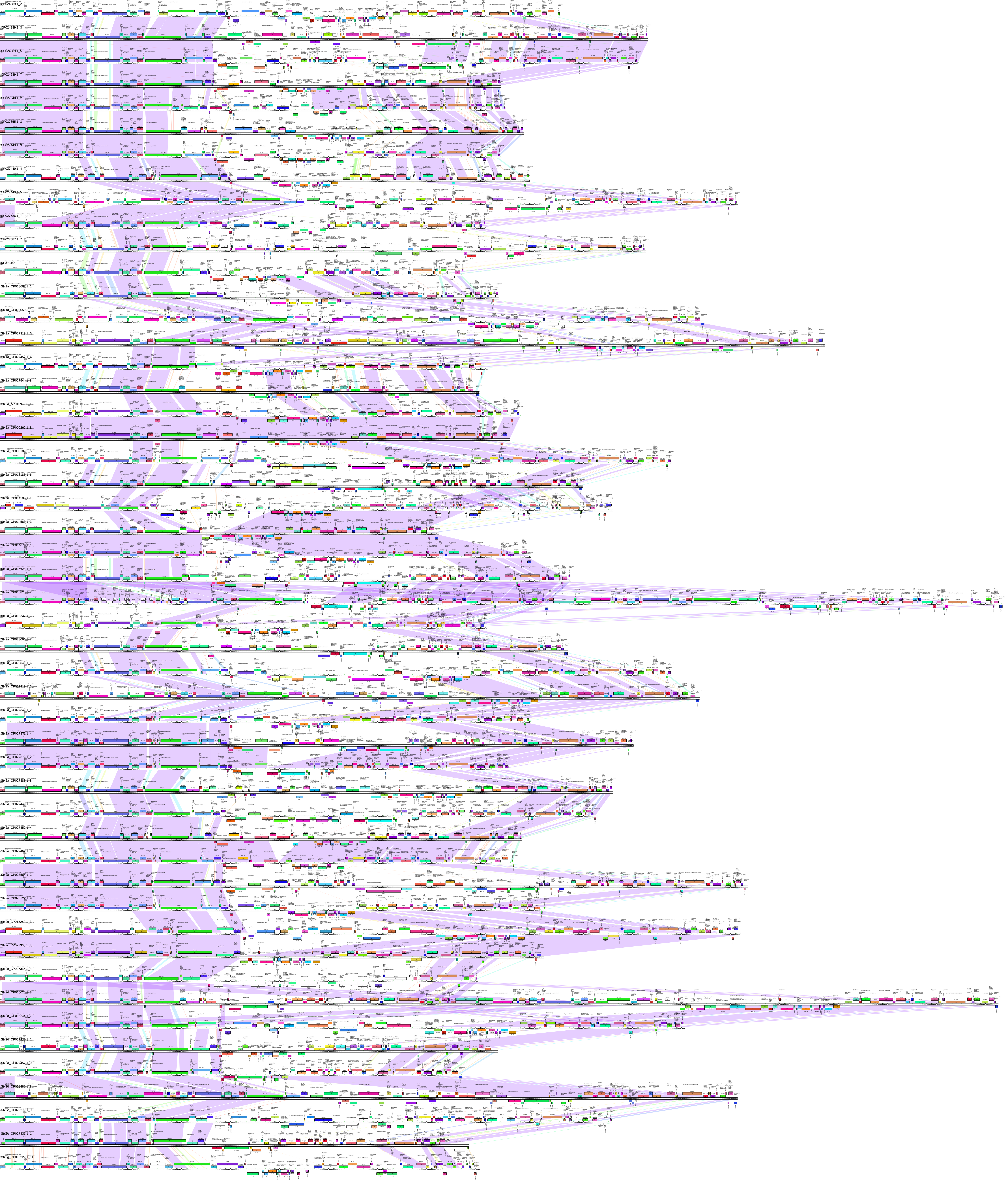

Supplement: Supplementary file 6 — Additional file 6. Whole-genome map of subcluster A2 phages. Represented as in Additional file 5. [file 12864_2021_7685_MOESM6_ESM.pdf]
