# Supplementary material for: Insights into the genome architecture and evolution of Shiga toxin encoding bacteriophages of Escherichia coli
Source: BMC Genomics. 2021 May 19;22:366. doi: 10.1186/s12864-021-07685-0 (PMC8136144; doi:10.1186/s12864-021-07685-0)
Supplement: Supplementary file 9 — Additional file 9. Whole-genome map of cluster C phages. Represented as in Additional file 5. [file 12864_2021_7685_MOESM9_ESM.pdf]

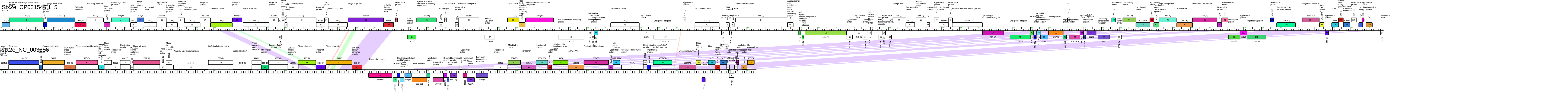
stx2e_CP031546.1_5
stx2e_NC_003356
