# Supplementary material for: Insights into the genome architecture and evolution of Shiga toxin encoding bacteriophages of Escherichia coli
Source: BMC Genomics. 2021 May 19;22:366. doi: 10.1186/s12864-021-07685-0 (PMC8136144; doi:10.1186/s12864-021-07685-0)
Supplement: Supplementary file 12 — Additional file 12. Lysis cassette map. The map was constructed using Phamerator using six randomly Stx phages as an example. Genes are labelled with their putative function, and phams with same predicted functional are represented by same color. Similarity is given by purple lines (minimal BLASTN cut-off E value is 10− 4). [file 12864_2021_7685_MOESM12_ESM.pdf]

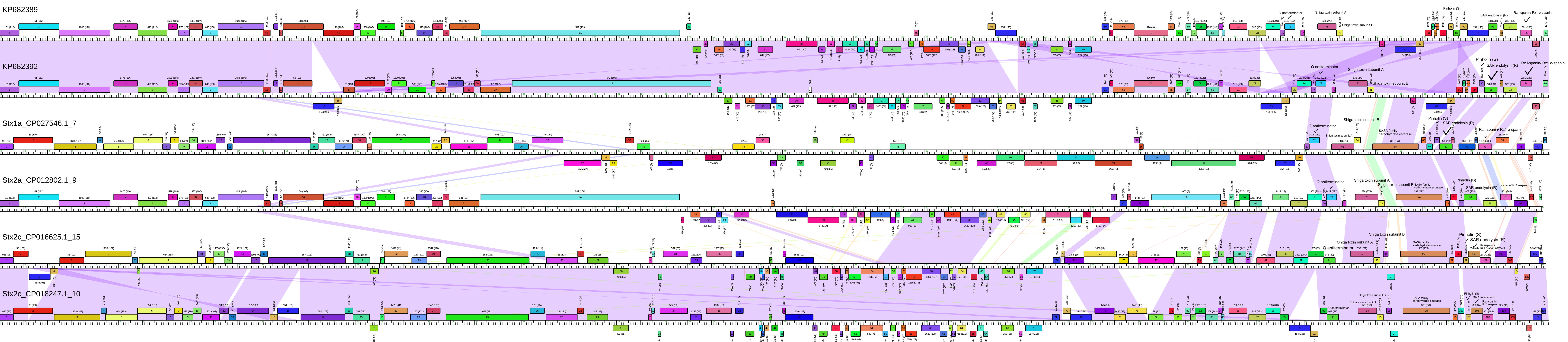

KP682389
KP682392
Stx1a_CP027546.1_7
Stx2a_CP012802.1_9
Stx2c_CP016625.1_15
Stx2c_CP018247.1_10
Q antiterminator
Shiga toxin subunit A
Shiga toxin subunit B
Pinholin (S)
SAR endolysin (R)
Rz i-spanin/ Rz1 o-spanin
SASA family carbohydrate esterase
